# Oral Antidiabetics and Sleep Among Type 2 Diabetes Patients: Data From the UK Biobank

**DOI:** 10.3389/fendo.2021.763138

**Published:** 2021-11-03

**Authors:** Pei Xue, Jiafei Wu, Xiangdong Tang, Xiao Tan, Christian Benedict

**Affiliations:** ^1^ Department of Neuroscience (Sleep Science, Biomedicinskt centrum (BMC)), Uppsala University, Uppsala, Sweden; ^2^ Sleep Medicine Center, Department of Respiratory and Critical Care Medicine, Mental Health Center, Translational Neuroscience Center, and State Key Laboratory of Biotherapy, West China Hospital, Sichuan University, Chengdu, China; ^3^ Department of Clinical Neuroscience, Karolinska Institutet, Solna, Sweden

**Keywords:** type 2 diabetes, metformin, sulphonylurea, insomnia, sleep duration, UK Biobank

## Abstract

Previous small-scale studies have found that oral antidiabetic therapy is associated with sleep difficulties among patients with type 2 diabetes (T2D). Here, we used data from 11 806 T2D patients from the UK Biobank baseline investigation to examine the association of oral antidiabetic therapy with self-reported difficulty falling and staying asleep and daily sleep duration. As shown by logistic regression adjusted for, e.g., age, T2D duration, and HbA_1c_, patients on non-metformin therapy (N=815; 86% were treated with sulphonylureas) had a 1.24-fold higher odds ratio of reporting regular difficulty falling and staying asleep at night compared to those without antidiabetic medication use (N=5 366, P<0.05) or those on metformin monotherapy (N=5 625, P<0.05). Non-metformin patients reported about 8 to 10 minutes longer daily sleep duration than the other groups (P<0.05). We did not find significant differences in sleep outcomes between untreated and metformin patients. Our findings suggest that non-metformin therapy may result in sleep initiation and maintenance difficulties, accompanied by a small but significant sleep extension. The results of the present study must be replicated in future studies using objective measures of sleep duration and validated questionnaires for insomnia. Considering that most T2D patients utilize multiple therapies to manage their glycemic control in the long term, it may also be worth investigating possible interactions of antidiabetic drugs on sleep.

## Introduction

Type 2 diabetes (T2D) patients often complain about sleep difficulties (1). For instance, about 77% regularly report insomnia symptoms, such as difficulty falling and staying asleep (2). According to the International Classification of Sleep Disorders criteria (3), having trouble falling asleep at night and waking up in the middle of the night constitute key insomnia symptoms. In addition to insomnia, T2D patients often report habitual short (usually defined as <7 hours per day) or long sleep duration (often described as >9 hours per day) (4).

Several factors may account for sleep difficulties among T2D patients, including but not limited to comorbidities such as overweight and hypertension (1). As suggested by previous studies, the type of oral antidiabetic agent used to prevent hyperglycemia may also account for sleep difficulties among T2D patients (5–7). For example, by using one-night sleep polysomnography, a study among 387 outpatients with T2D found that those on metformin slept on average about 36 minutes longer and exhibited a 6.4% higher sleep efficiency compared to patients on non-metformin (mainly sulfonylureas) (5). Metformin, a biguanide, suppresses hepatic glucose production and intestinal glucose absorption and promotes β-cell functions and insulin sensitivity (8–10). Sulfonylureas lower blood glucose by stimulating insulin release from pancreatic β-cells (11, 12).

Besides the mechanism of action, oral antidiabetics can also differ in the type, frequency, and severity of side effects (13–19). For instance, hypoglycemia represents the most common side effect of sulphonylureas (17). Diarrhea, nausea, vomiting, abdominal bloating, abdominal cramping or pain, flatulence, and anorexia, occur in approximately 10% to 30% of metformin recipients (18, 19). Side effects caused by oral antidiabetic therapy may lead to sleep difficulties, primarily if occurring during sleep.

In the present study, we wanted to examine the association of oral antidiabetics with sleep among T2D patients from the UK Biobank. Specifically, we used cross-sectional data from 11 806 T2D patients to determine whether the ability to initiate or maintain sleep and habitual sleep duration would vary by the type of oral antidiabetic agent used. 

## Materials and Methods

### Study Design and Participants

From 2006 to 2010, the UK Biobank recruited about 500 000 individuals. For the present study, we used the two following criteria to identify T2D patients: (a) we used a validated algorithm based on self-reported disease, medication, and T2D diagnosis in medical history (20); and (b) the patient used glucose-lowering medication and had an hemoglobin A_1c_ (HbA_1c_) level ≥6.5% (48mmol/mol). Records on antihyperglycemic medication derived from the UK Biobank interview. We assigned patients to one of the following three groups: not treated with oral antidiabetics, treated with metformin, and being on non-metformin therapy. We did not consider patients eligible for the analysis when they were on two or more oral antidiabetics (e.g., metformin and sulphonylurea) or insulin therapy. We also excluded individuals with probable type 1 diabetes or gestational diabetes identified by the diabetes algorithm (20). Following further exclusions as detailed in **Table 1**, 11 806 T2D patients represented our final sample.

**Table 1:** Exclusions.

	Number of subjects
Initial cohort	502 543
T2D patients	27 370
Treated with two or more oral antidiabetic medications	5 626
Treated with insulin	3 131
No information regarding insomnia (dependent variable)	95
No information regarding sleep duration (dependent variable)	240
No information regarding snoring (confounder)	1 591
No information regarding BMI (confounder)	132
No information regarding systolic blood pressure (confounder)	35
No information regarding HbA_1c_ (confounder)	1 024
No information regarding diabetes duration (confounder)	3 669
No information regarding Townsend index (confounder)	21
**Final cohort**	**11 806**

T2D, type 2 diabetes; BMI, body mass index; HbA_1c_, hemoglobin A_1c_.

The UK Biobank study was approved by the National Health Service National Research Ethics Service (ref. 11/NW/0382) and all participants provided written informed consent to participate in the UK Biobank study. Information about ethics oversight in the UK Biobank can be found at https://www.ukbiobank.ac.uk/ethics/.

### Outcomes

Habitual sleep duration was estimated by the question “About how many hours sleep do you get every 24 hours? (please include naps)”. Patients were also asked, “Do you have trouble falling asleep at night or do you wake up in the middle of the night?”. They had the following response options: “never/rarely”, “sometimes,” and “usually.” For the logistic regression analysis, the response option “usually” was defined as an event, i.e., the patients often complained about having trouble falling asleep at night or waking up in the middle of the night.

### Confounders

Confounders were selected based on our *a priori* knowledge of the relationships among potential confounders, intermediate variables, and outcome variables. Age (continuous scale), sex, region of UK Biobank assessment center, Townsend deprivation index reflecting a person’s socioeconomic status (continuous scale), BMI (continuous scale), and systolic blood pressure (continuous scale) derived from the UK Biobank reception information, baseline characteristics, touchscreen questionnaire, or physical measurement. Obstructive sleep apnea (OSA) represents common comorbidity of T2D (21), which can affect both sleep quality and duration (22). One of OSA’s cardinal symptoms is snoring (22). Thus, in the present study, we controlled for self-reported snoring. Snoring was assessed by the following question: “Does your partner or a close relative or friend complain about your snoring?” Patients had the four following response options: “Yes,” “No,” “Don’t know,” or “Prefer not to answer.” For the analysis, we excluded those responding, “Don’t know” or “Prefer not to answer” (**Table 1**). The duration of T2D (continuous scale) was calculated by subtracting self-reported age at T2D diagnosis from the age when the participant attended the UK Biobank baseline investigation. We also controlled our analysis for the use of antidepressants (selective serotonin reuptake inhibitors, serotonin-norepinephrine reuptake inhibitors, and tricyclic antidepressants) and statins, as both can influence sleep (23, 24). In addition, we also retrieved data on the use of sedatives, such as barbiturates, benzodiazepines, Z-drugs, antihistamines, and other sedative medications. Finally, information about T2D comorbidities and complications was retrieved from the UK Biobank interview. Due to the low prevalence of sedative use, stroke, chronic kidney disease, and T2D complications, these variables were not considered as confounders in the logistic regression analysis.

Findings from previous studies suggest that glycemic control, often assessed by the HbA_1c_, is inversely associated with sleep quality and duration (25–27). Thus, we also controlled our analysis for patients’ HbA_1c_. The HbA_1c_ (continuous scale) was determined with high-performance liquid chromatography using the Bio-Rad VARIANT II TURBO HbA_1c_ analyzer.

### Statistical Analysis

We performed all analyses with IBM SPSS version 24.0 (Inc., Chicago, IL, USA). Baseline characteristics among the three treatment groups were compared with univariate generalized linear models and chi-square tests. We used logistic regression analysis to compare the odds ratio of having trouble falling asleep at night or waking up in the middle of the night among the three groups. Finally, we explored possible group differences in daily sleep duration by generalized linear models. Overall, a p-value smaller than 0.05 was considered significant.

## Results

**Figure 1:**
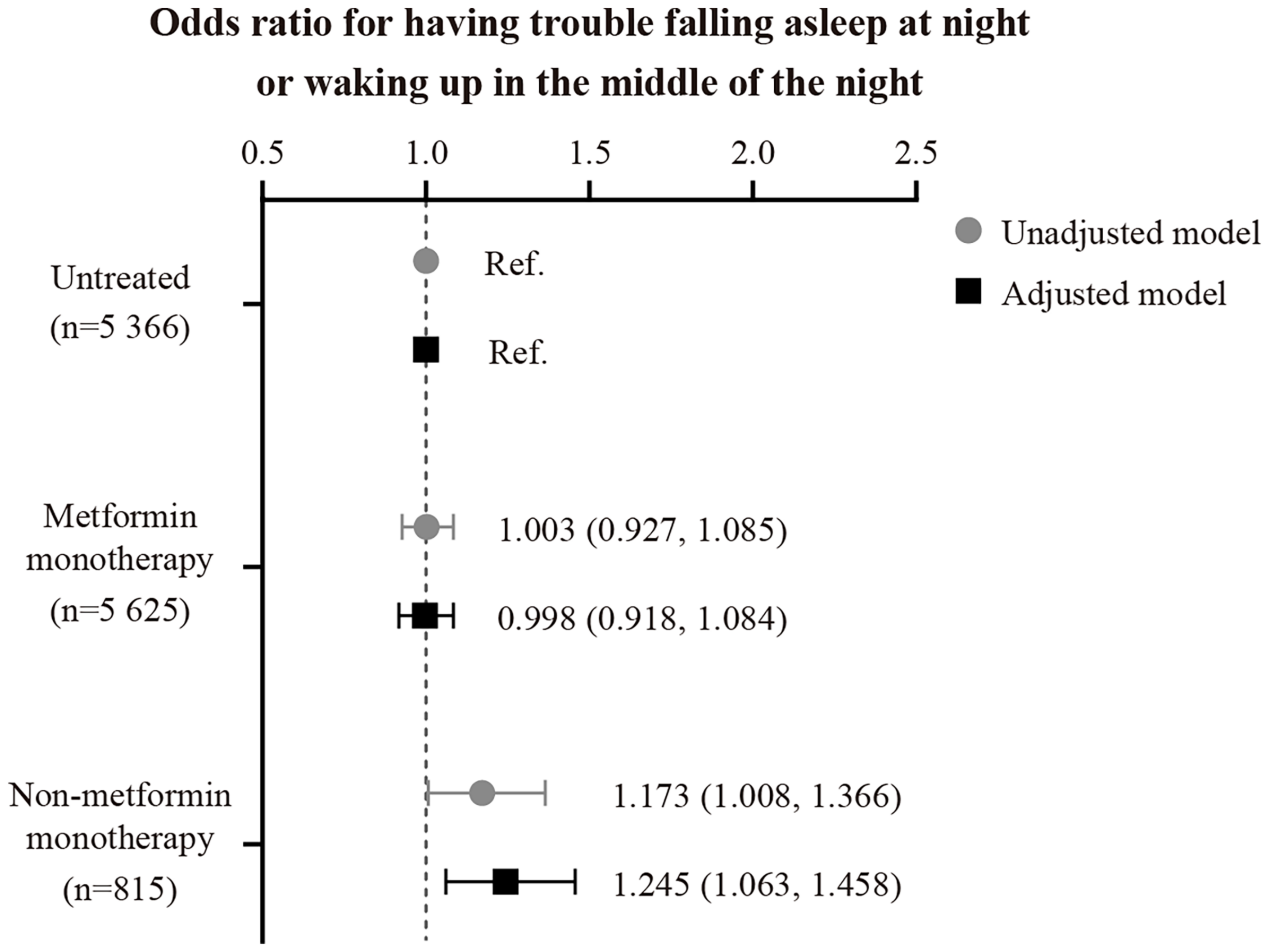
Unadjusted and adjusted odds ratios and 95% CIs for having trouble falling asleep at night or waking up in the middle of the night, stratified by the oral antidiabetic regimen. The adjusted model was controlled for patients’ age, sex, BMI, UK Biobank assessment center, Townsend index, systolic blood pressure, snoring status, type 2 diabetes duration, HbA_1c_, presence of cardiovascular disease, antidepressant therapy status, and statin therapy status.

**Figure 2:**
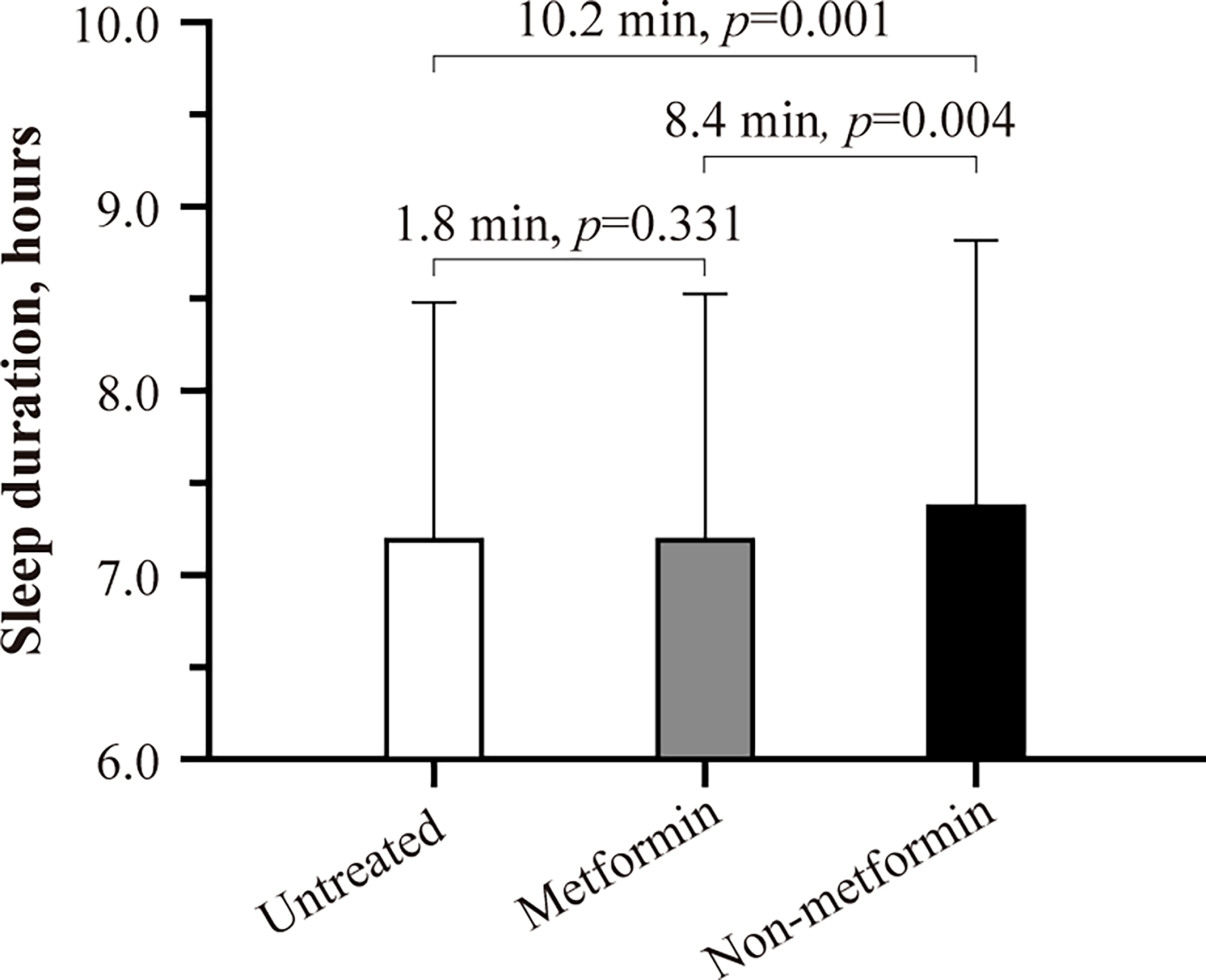
Habitual sleep duration of untreated, metformin, and non-metformin patients with type 2 diabetes. Estimated marginal means ± SE derived from a generalized linear model adjusting for patients’ age, sex, BMI, UK Biobank assessment center, Townsend index, systolic blood pressure, snoring status, type 2 diabetes duration, HbA_1c_, presence of cardiovascular disease, antidepressant therapy status, and statin therapy status are shown. P smaller than 0.05 was considered significant.

Baseline characteristics are summarized in **Table 2**. In the full cohort (n=11 806), ~35% of the patients reported that they usually struggle with falling or staying asleep. As shown in the fully-adjusted logistic regression (**Figure 1**), patients using non-metformin agents (n=815) had a 1.24-fold higher odds ratio of reporting regular difficulty falling and staying asleep at night than patients without antidiabetic medication use (n=5 366; P=0.007). Additional multivariable analysis revealed that patients using non-metformin had higher odds of having trouble falling and staying asleep when using metformin patients as the reference group (n=5 625) (OR[95%-CI], 1.244 [1.064,1.455]; P=0.006). Non-metformin patients also reported longer sleep duration than untreated and metformin patients (P=0.004 for the main effect of treatment group; derived from the fully-adjusted generalized linear model; see **Figure 2** for group comparisons). Comparisons between metformin patients and those without antidiabetic medication use did not reveal significant group differences (**Figures 1**, **2**).

**Table 2:** Baseline characteristics of untreated, metformin, and non-metformin patients with type 2 diabetes.

Characteristic	Oral antidiabetic regimen			*P* value
	Untreated (n = 5 366)	Metformin (n = 5 625)	Non-metformin (n = 815)	
Age at investigation, mean (SD), y	60.7 (6.6)	59.8 (6.9)	61.3 (6.7)	<.001^†^
Male sex	3 438 (64.1)	3 566 (63.4)	600 (73.6)	<.001^#^
Difficulty falling and staying asleep	1 843 (34.3)	1 936 (34.4)	310 (38.0)	.106^#^
Sleep duration, mean (SD), h	7.19 (1.29)	7.19 (1.34)	7.37 (1.45)	.001^†^
Snoring	2 573 (48.0)	2 635 (46.8)	365 (44.8)	.182^#^
BMI, mean (SD), kg/m^2^	31.0 (5.4)	31.7 (5.6)	30.5 (5.6)	<.001^†^
Systolic blood pressure, mean (SD), mmHg	145 (19)	143 (18)	144 (19)	<.001^†^
HbA1c, mean (SD), mmol/mol	45.8 (10.2)	51.6 (11.7)	53.2 (13.2)	<.001^†^
Duration of type 2 diabetes, mean (SD), y	4.6 (7.6)	5.7 (7.9)	8.3 (9.0)	<.001^†^
Region of assessment center				
England	4 696 (87.5)	5 091 (90.5)	695 (85.3)	<.001^#^
Wals	283 (5.3)	214 (3.8)	32 (3.9)	
Scotland	387 (7.2)	320 (5.7)	88 (10.8)	
Townsend index, mean (SD)	-0.89 (3.26)	-0.46 (3.37)	-0.46 (3.51)	<.001^†^
Other medication				
Antidepressant use	163 (3.0)	162 (2.9)	22 (2.7)	.812^#^
Statin use	258 (4.8)	174 (3.1)	25 (3.1)	<.001^#^
Sedatives use	3 (0.1)	1 (0.02)	1 (0.1)	–
Type 2 diabetes comorbidities				
CVD (I20, I21, I25, I48 and I50)*	102 (1.9)	113 (2.0)	28 (3.4)	.015^#^
Stroke (I60, I61, I63, I64)*	12 (0.2)	16 (0.3)	6 (0.7)	–
Chronic kidney Disease (N03, N11, N18)*	2 (0.04)	0	1 (0.1)	–
Type 2 diabetes complication	3 (0.1)	13 (0.2)	3 (0.4)	–
Type of oral antidiabetic monotherapy				
Metformin	–	100	–	–
Sulfonylurea	–	–	85.9	–
Glitazone	–	–	12.8	–
Meglitinide	–	–	1.2	–
Other oral antidiabetics	–	–	0.1	–

BMI, body mass index; HbA_1c_, hemoglobin A_1c_; CVD, cardiovascular disease.

Data are presented as number (percentage) of study participants unless otherwise indicated.

^†^Univariate generalized linear model.

^#^Chi-square tests.

*Referring to ICD-10 codes.

## Discussion

There is evidence that oral antidiabetics may impact sleep; however, with mixed results. One study reported longer and more efficient sleep among T2D patients on metformin than those using non-metformin agents (5). In contrast, two other studies found that metformin may be associated with poor sleep quality and frequent nightmares (6, 7). In the present study, including 11 806 patients with T2D, we found that those treated with non-metformin were more likely to report difficulty falling and staying asleep at night compared to those without antidiabetic medication use or those on metformin monotherapy. More than four out of five patients in the non-metformin group were on sulfonylurea monotherapy. Hypoglycemia, the most common side effect of sulphonylureas (17), can induce counterregulatory stress hormone responses (28). If occurring at bedtime or during sleep, these hypoglycemia-induced endocrine stress responses may cause hyperarousal and, therefore, impair the ability of the patient to fall and stay asleep. However, due to the lack of hypoglycemia screening in the UK Biobank, this explanation remains speculative. We also found that patients on non-metformin slept about 8 to 10 minutes longer than those on metformin or without antidiabetic treatment. Whether the small sleep extension was due to accompanying sleep initiation and maintenance difficulties among non-metformin patients is unclear.

Several limitations apply to the present study. It remains unclear whether factors such as antidiabetic dosing and timing of medication administration may modulate the association of oral antidiabetics with sleep. Furthermore, the UK Biobank investigation did not survey adverse side effects of oral antidiabetics, which could explain differences in sleep outcomes between metformin and non-metformin users. In T2D patients with low kidney function, physicians often prescribe sulfonylureas (29). Chronic kidney disease has been linked to poor sleep quality (30). Importantly, only three of 11 806 patients had chronic kidney disease at the baseline investigation in the present study. Thus, it is unlikely that the observed sleep difficulties among patients treated with non-metformin are attributable to chronic kidney disease. Furthermore, in the present study, only a few patients reported the use of sleep-promoting sedatives. However, we could not control our analysis for the possible use of sleep-promoting over-the-counter-remedies (e.g., melatonin). The present study was further limited by the small sample size of patients treated with non-metformin drugs. Thus, future studies with larger sample sizes, including novel types of non-metformin oral antidiabetics than those investigated herein (e.g., dipeptidyl-peptidase 4 inhibitors, glucagon-like peptide-1 receptor agonists), are needed to advance our understanding of how oral antidiabetics impact sleep among patients with T2D. Considering that most T2D patients utilize multiple therapies to manage their glycemic control in the long term, it may also be worth investigating possible interactions of antidiabetic drugs on sleep. Finally, sleep duration was derived from self-reports, which might be subject to potential recall bias. Thus, our results must be replicated in studies using longitudinal measures of sleep duration (e.g., sleep-tracking wearables) and validated questionnaires for insomnia (e.g., insomnia severity index).

Despite these limitations, the primary strength of our study is that our results were based on one of the largest cohorts worldwide. Furthermore, findings were robust to adjustment for important confounders, such as snoring, age, BMI, blood pressure, and HbA_1c_.

## Conclusions

Recurrent problems with falling and staying asleep have been associated with impaired glycemic control among patients with T2D (31–33). To mitigate these possible adverse consequences, T2D patients on non-metformin should regularly participate in sleep screenings. As shown herein, they more often suffer from sleep initiation and maintenance difficulties.

## Data Availability Statement

The datasets presented in this article are not readily available because data may be obtained from a third party and are not publicly available. Data were derived from the UK Biobank investigation. Thus, data may be obtained from the UK Biobank upon request. Requests to access the datasets should be directed to https://www.ukbiobank.ac.uk/.

## Ethics Statement

The studies involving human participants were reviewed and approved by National Health Service National Research Ethics Service (ref. 11/NW/0382). The patients/participants provided their written informed consent to participate in this study.

## Author Contributions

CB and PX designed the study. PX and JW performed analysis. CB and PX drafted the manuscript. All authors interpreted the results and critically revised the manuscript for intellectual content. PX takes responsibility for the accuracy of the data analysis. All authors approved the final version of this manuscript to be published.

## Funding

The authors’ work is funded by the Novo Nordisk Foundation (CB; NNF19OC0056777), Swedish Brain Research Foundation (CB; FO2020-0044), Åke Wiberg Foundation (XiaoT; M18-0169, M19-0266), Fredrik and Ingrid Thuring Foundation (XiaoT; 2019-00488), and the Swedish Society for Medical Research (XiaoT). The study sponsor/funder was not involved in the design of the study; the collection, analysis, and interpretation of data; writing the report; and did not impose any restrictions regarding the publication of the report.

## Conflict of Interest

The authors declare that the research was conducted in the absence of any commercial or financial relationships that could be construed as a potential conflict of interest.

## Publisher’s Note

All claims expressed in this article are solely those of the authors and do not necessarily represent those of their affiliated organizations, or those of the publisher, the editors and the reviewers. Any product that may be evaluated in this article, or claim that may be made by its manufacturer, is not guaranteed or endorsed by the publisher.
